# Determinants of disparities of diabetes-related hospitalization rates in Florida: a retrospective ecological study using a multiscale geographically weighted regression approach

**DOI:** 10.1186/s12942-023-00360-5

**Published:** 2024-01-06

**Authors:** Jennifer Lord, Agricola Odoi

**Affiliations:** Department of Biomedical and Diagnostic Sciences, College of Veterinary Medicine, The University of Tennessee, Knoxville, TN USA

**Keywords:** Diabetes, Hospitalizations, Multiscale geographically weighted regression, Geographic Information Systems

## Abstract

**Background:**

Early diagnosis, control of blood glucose levels and cardiovascular risk factors, and regular screening are essential to prevent or delay complications of diabetes. However, most adults with diabetes do not meet recommended targets, and some populations have disproportionately high rates of potentially preventable diabetes-related hospitalizations. Understanding the factors that contribute to geographic disparities can guide resource allocation and help ensure that future interventions are designed to meet the specific needs of these communities. Therefore, the objectives of this study were (1) to identify determinants of diabetes-related hospitalization rates at the ZIP code tabulation area (ZCTA) level in Florida, and (2) assess if the strengths of these relationships vary by geographic location and at different spatial scales.

**Methods:**

Diabetes-related hospitalization (DRH) rates were computed at the ZCTA level using data from 2016 to 2019. A global ordinary least squares regression model was fit to identify socioeconomic, demographic, healthcare-related, and built environment characteristics associated with log-transformed DRH rates. A multiscale geographically weighted regression (MGWR) model was then fit to investigate and describe spatial heterogeneity of regression coefficients.

**Results:**

Populations of ZCTAs with high rates of diabetes-related hospitalizations tended to have higher proportions of older adults (*p* < 0.0001) and non-Hispanic Black residents (*p* = 0.003). In addition, DRH rates were associated with higher levels of unemployment (*p* = 0.001), uninsurance (*p* < 0.0001), and lack of access to a vehicle (*p* = 0.002). Population density and median household income had significant (*p* < 0.0001) negative associations with DRH rates. Non-stationary variables exhibited spatial heterogeneity at local (percent non-Hispanic Black, educational attainment), regional (age composition, unemployment, health insurance coverage), and statewide scales (population density, income, vehicle access).

**Conclusions:**

The findings of this study underscore the importance of socioeconomic resources and rurality in shaping population health. Understanding the spatial context of the observed relationships provides valuable insights to guide needs-based, locally-focused health planning to reduce disparities in the burden of potentially avoidable hospitalizations.

**Supplementary Information:**

The online version contains supplementary material available at 10.1186/s12942-023-00360-5.

## Highlights

- Diabetes-related hospitalization rates exhibited marked variation at the local level, which may be masked in investigations of larger geographic units. Hospitalization rates can be a useful indicator of diabetes outcomes at the local level, particularly in states or countries without population-level data from disease registries and/or spatially representative health surveys.
- This is the first study to use multiscale geographically weighted regression (MGWR) to investigate determinants of diabetes-related hospitalization rates at the local level. Strengths of associations between determinants and hospitalization rates varied based on geographic location within the study area. This information is useful to guide targeted resource allocation and needs-based health planning, and MGWR can be employed in other study areas to investigate spatially-variable determinants of diabetes-related outcomes.
- Associations between the identified determinants and diabetes-related hospitalization rates exhibited spatial heterogeneity at local, regional, and statewide levels. This information can serve policymakers and public health planners by suggesting the spatial scale at which a given intervention strategy should be implemented. Future ecological studies should consider spatial scale as well as geographic location when investigating determinants of diabetes and other chronic disease outcomes.

**Supplementary Information:**

The online version contains supplementary material available at 10.1186/s12942-023-00360-5.

## Background

In the United States, the prevalence of diabetes among adults is estimated to be as high as 14.7%, and is projected to increase to 17.9% by 2060 [1, 2]. Early diagnosis, control of blood glucose levels and cardiovascular risk factors, and regular screening for complications are key components of diabetes care to prevent or delay complications and reduce the risk of major clinical events that necessitate hospitalization [3–6]. However, fewer than 1 in 4 US adults with diagnosed diabetes meet recommended targets for glucose, blood pressure, and lipid levels [7]. Despite prior improvements in achieving these goals, the percentage of adults with diabetes meeting glycemic and blood pressure targets have declined since the early 2010s, while the percentage with lipid control has plateaued [7–9]. In addition, rates of lower extremity amputations due to diabetes and diabetes-associated hospitalizations in the US have increased over the course of the past decade [10, 11]. In 2017, $69.7 billion in healthcare expenditures in the US were attributed to hospital inpatient stays by patients with diabetes [12].

There is evidence of geographic disparities in access and utilization of diabetes care, as well as burden of diabetes and diabetes-related complications in the United States [13–17]. A contiguous region in the Southeastern US has been termed the Diabetes Belt due to the disproportionately high diabetes prevalence in this region compared to the rest of the country [13, 14]. However, despite the relatively high burden of the condition, those with diabetes in the Southern US have lower odds of receiving hemoglobin A1c (HbA1c) testing at recommended intervals, and higher odds of foregoing necessary medical care due to cost compared to other regions [15, 16]. In addition, patients with diabetes in the South have higher odds of experiencing hypoglycemic events, and higher odds of death during a diabetes-related hospitalization compared to other parts of the country [15, 17].

In Florida, the most populous state in the Southeastern US, over 2 million adults have been diagnosed with diabetes [18], and it is estimated that an additional 546,000 Floridians have diabetes that is yet to be diagnosed [19]. Within the state, there is significant geographic variation in the distribution of diabetes prevalence [20–22]. In addition, recent research has identified local geographic hotspots of diabetes-related hospitalization (DRH) rates [23], indicating that some communities in the state bear a disproportionately high burden of potentially preventable diabetes complications. Communities in these hotspots also face a major financial burden; in 2018, the median cost of a hospital stay due to diabetes in Florida was $40,718 [24]. In order to reduce the observed disparities in DRH rates, it is important to close gaps in adherence to preventive practices and utilization of diabetes care [7, 8]. Identifying predictors of hospitalization rates will provide useful information to guide public health planning and evidence-based interventions to address barriers to achieving diabetes care targets. Furthermore, understanding the spatial context of these relationships can help guide resource allocation and ensure that future interventions are designed to meet the specific needs of these communities. Therefore, the objectives of this study were (1) to identify determinants of diabetes-related hospitalization rates at the ZIP code tabulation area (ZCTA) level in Florida, and (2) assess if the strengths of these relationships vary by geographic location and at different spatial scales.

## Methods

### Study design and setting

**Fig. 1:**
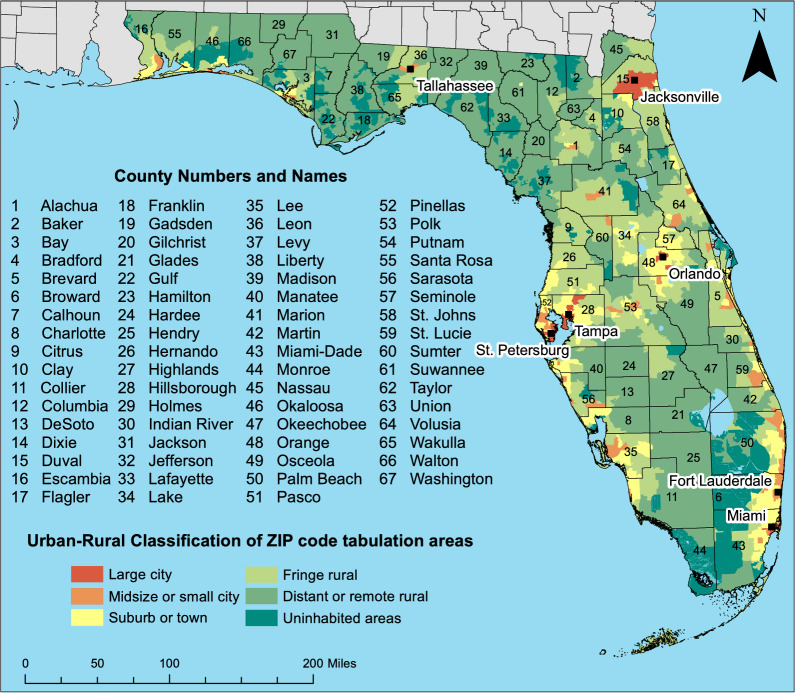
Geographic distribution of counties and major cities and urban–rural classification of ZCTAs in Florida, USA

This retrospective ecological study was conducted in the Southeastern US state of Florida. In 2019, the state had an estimated adult population of 16.7 million, 4.2 million of whom were aged 65 and older [25]. There are 983 ZIP code tabulation areas (ZCTAs), which are areal representations of post office ZIP codes, in Florida (Fig. 1) [26]. In 2019, the estimated diabetes prevalence among Florida adults was 11.7%, compared to 9.3% for the US overall [27]. However, prevalence estimates at the ZCTA level in Florida ranged from 1.0% to 24.5% [28]. The main outcome measure in this study was ZCTA-level diabetes-related hospitalization (DRH) rates among adults 18 years of age and older during the period from 2016 to 2019. Factors investigated for potential associations with DRH rates were socioeconomic, demographic, healthcare-related, and built environment characteristics.

### Data sources

Hospital inpatient data files were provided by the Florida Agency for Health Care Administration through a Data Use Agreement with the Florida Department of Health. All other data used in the study were obtained from publicly available sources. Model-based small area estimates of diabetes prevalence were obtained from the Centers for Disease Control and Prevention (CDC) Population-Level Analysis and Community Estimates (PLACES) project [29]. Population size, socioeconomic and demographic characteristics were obtained from the US Census Bureau American Community Survey 5-year estimates [30–35]. Built environment variables assessed included food retailers and fitness/recreational facilities. Data on these variables were obtained from the 2016 US Census Bureau ZIP Code Business patterns [36]. Address ZIP codes for pharmacies and practices of internal medicine and family practice physicians were obtained from the Florida Department of Health, Division of Medical Quality Assurance’s Florida Healthcare Practitioner Data Portal [37]. Locale assignments for rural–urban classification of ZCTAs were obtained from the National Center for Education Statistics [38]. Cartographic boundary shapefiles used for mapping were downloaded from the U.S. Census Bureau TIGER Geodatabase and the Florida Geographic Data Library [39, 40].

### Data preparation

#### Diabetes-related hospitalizations

Records of diabetes-related hospitalizations for patients aged 18 to 100 years with home address ZIP codes in Florida and admission dates between January 1, 2016 and December 31, 2019 were extracted from the hospital discharge data. The inclusion criteria for diabetes-related hospitalizations for this study were based on the Agency for Healthcare Research and Quality (AHRQ) Prevention Quality Indicators, the Diabetes Complications Severity Index (DCSI), and reporting by the Florida Diabetes Advisory Council [24, 41–46]. Diabetes-related hospitalizations were defined as those with: a principal diagnosis code for diabetes with short-term complications, long-term complications, or uncontrolled diabetes [41–43]; a procedure code for lower-extremity amputation (including toe amputations) in any field and a diagnosis of Type 1 or Type 2 diabetes mellitus in any field [24, 44]; or a principal diagnosis of a diabetes complication included in the DCSI and a diagnosis of Type 1 or Type 2 diabetes mellitus in any field [45, 46].

Patient address ZIP codes were joined to ZCTAs using the crosswalk table developed by John Snow, Inc., and aggregated to the ZCTA level [47]. Rates of diabetes-related hospitalizations were computed by dividing the number of diabetes-related hospitalizations by the total number of person-years at risk. The median age of patients with diabetes-related hospitalizations for each ZCTA was also computed. After exclusion of ZCTAs with fewer than 10 diabetes-related hospitalizations or populations of fewer than 100 adult residents to minimize disclosure risk and small number bias, 933 ZCTAs remained for further analysis.

#### Socioeconomic, demographic, healthcare-related and built environment variables

The following socioeconomic and demographic variables were considered as potential predictor variables: population density, median age, median household income and household value, percent of the population that were 65 years or older, Hispanic or non-Hispanic Black, 25 years or older without a high school education, 25 years or older with a bachelor’s degree or higher, percent of families with income below the poverty level, percent of unemployed residents, percent without health insurance coverage, and percent of households without a vehicle.

North American Industry Classification System (NAICS) codes were used to extract the number of businesses in each ZIP code classified as: convenience stores (NAICS code 445120), limited service restaurants (NAICS code 722211), supermarkets (NAICS code 445110), fruit and vegetable markets (NAICS code 445230), warehouse clubs (NAICS 452910), and fitness and recreational sports centers (NAICS 713940). The John Snow, Inc. crosswalk was used to join ZIP codes of food retailers, fitness/recreational centers, pharmacies, and primary care practices to ZCTAs [47]. A ZCTA-level version of the modified retail food environment index (mRFEI), which measures the percent of “healthy” food retailers in an area [48], was also computed. Businesses included in the category of healthy food retailers were supermarkets, produce markets, and warehouse clubs, while those included in the “less healthy” category included limited service (fast food) restaurants and convenience stores. ZCTA-level densities of food retailers, fitness/recreational facilities, and pharmacies were computed using land area from the ZCTA shapefile. The number of primary care practices per 10,000 population in each ZCTA was also computed.

### Statistical analysis

#### Descriptive statistical analyses

Statistical analysis was performed using SAS version 9.4 [49]. Normality of distribution of continuous ZCTA-level characteristics was assessed using the Shapiro–Wilk test, and median and interquartile range (IQR) were used as measures of central tendency and dispersion for non-normally distributed variables. Characteristics of hospitalizations and ZCTAs included in the spatial analysis were compared with those that did not meet the inclusion criteria using the Wilcoxon rank sum test for continuous variables, and the Chi-square test for categorical variables. Diabetes-related hospitalization rates were smoothed in GeoDa 1.18.0 using the spatial empirical Bayes approach with queen contiguity weights [50].

#### Ordinary least squares regression model

An ordinary least squares (OLS) regression model was fit to the data to identify predictors of diabetes-related hospitalization rates. Due to the highly skewed distribution of ZCTA-level DRH rates, natural log transformation of the outcome measure was performed. In addition, all continuous variables were standardized to have a mean of 0 and standard deviation of 1 to facilitate comparison with the results of the MGWR analysis described in the following section. To identify candidate variables for inclusion in the multivariable model-building process, univariable associations between potential predictors and log-transformed DRH rates were first assessed. A significance level of 0.15 was used to identify variables for consideration in the multivariable model.

To prevent problems with multicollinearity, Spearman’s rank correlation coefficient was used to identify pairs of highly correlated (|r_s_|≥ 0.7) variables, from which one variable was selected as a potential predictor for inclusion in the model-building process. Variable selection for the multivariable OLS regression model was performed using manual backwards elimination, using a critical *p*-value of 0.05. During the model-building process, collinearity between explanatory variables was assessed using variance inflation factor (VIF), with VIF ≥ 10 indicating multicollinearity [51]. Confounding was assessed using theoretical and empirical methods. Variables hypothesized to be confounders based on literature review were included in the model regardless of statistical significance. In addition, the magnitudes of changes in coefficient estimates were examined as each variable was removed from the model. A variable was considered for retention in the model regardless of statistical significance as a potential confounder if its removal resulted in a 20% or greater change in the coefficient estimate of another variable in the model and its inclusion did not substantially worsen model fit. Univariable OLS regression was also used to assess for association between log-transformed DRH rates and the median age of patients with diabetes-related hospitalizations.

#### Multiscale geographically weighted regression model

Global regression models assume that relationships between an outcome variable and its determinants do not vary by geographic location within a given study area [52]. However, violations of this assumption may occur with geographical data, resulting in model misspecification. Therefore, a multiscale geographically weighted regression (MGWR) analysis was performed to assess for spatial variability in regression coefficients and determine the optimal model to assess these relationships [53–56]. Rather than computing a single coefficient that estimates the average association between an explanatory and outcome variable for the entire study area, geographically weighted regression models estimate a regression coefficient for each location in the study area [52]. Model parameters for each geographic unit are estimated using a local subset of observations that are weighted by their distance from that location [52]. The weight assigned to a given observation depends on the type of weighting function selected, and its bandwidth determines the size of the local subset of observations used to estimate model parameters [52]. Multiscale GWR differs from standard and semiparametric GWR in that a unique kernel bandwidth is computed for each explanatory variable in the model [53–56]. In standard GWR, a single bandwidth is computed and used for all explanatory variables in the model [53–56]. Semiparametric GWR allows for specification of variables as either global (stationary) or local (spatially non-stationary), with a single bandwidth computed and used for all local variables in the model [53–56].

The multiscale GWR analysis was implemented using the *mgwr* package in Python, specifying the explanatory variables as those selected for the final global multivariable OLS model [57]. To enable comparison of bandwidth values, continuous variables were standardized to have a mean of 0 and standard deviation of 1 [53–57]. An adaptive bi-square weighting kernel was selected as the weighting scheme. This method is useful when geographic units within the study area vary in size, because the radius of the kernel depends on a fixed number of nearest neighbors rather than a fixed distance [53, 58]. Therefore, the optimal bandwidth for each variable may be interpreted as the number of nearest neighbors used to estimate its local regression coefficient for each ZCTA in the study area [53, 58]. Optimal bandwidths were selected using the Golden section search method and the bias-corrected Akaike’s Information Criterion (AICc) as the optimization criterion. Covariate-specific critical *t*-values, corrected for multiple testing by adjusting for the effective number of parameters, were used to identify ZCTAs with statistically significant relationships between each explanatory variable and the dependent variable [54, 57]. An explanatory variable was considered to exhibit significant spatial non-stationarity if the interquartile range of its coefficient estimates from the MGWR model was greater than twice the standard error of its coefficient estimate from the global OLS model [58].

The optimal bandwidths of the variables in the MGWR model were examined to determine whether a simpler, alternate GWR model should be considered [55, 56]. For example, spatial non-stationarity and similar bandwidths for all variables would suggest a standard GWR model may be appropriate [55, 56]. A subset of stationary variables, with the remainder exhibiting non-stationarity and having similar bandwidths, would suggest a semiparametric GWR model should be considered [55, 56]. Model fit of the global and local models was compared using AICc values. Residual plots were generated to assess the distribution of the standardized residuals from the final model, assess for heteroskedasticity, and identify outliers.

### Cartographic displays

Smoothed diabetes-related hospitalization rates and diabetes prevalence estimates obtained from CDC PLACES data were displayed in choropleth maps using ArcGIS [29, 59]. In addition, choropleth maps were generated to show the ZCTA-level distribution of explanatory variables from the final MGWR model and to display local coefficients for non-stationary variables. The class ranges used for display of continuous variables in map figures were determined using Jenks’ natural breaks classification scheme, except for population density, which was displayed using a quantile map [60].

## Results

### Descriptive analyses

There were 554,679 diabetes-related hospitalizations in Florida between 2016 and 2019. A total of 546 hospitalizations (0.098%) for patients from 50 ZCTAs that had fewer than 10 diabetes-related hospitalizations during the study period and/or a population at risk of fewer than 100 adult residents during any year of the study period were excluded. Characteristics of excluded hospitalizations and ZCTAs and comparisons with those that met the inclusion criteria are listed in Additional file 1: Table S1. In total, 554,133 diabetes-related hospitalizations from patients in 933 ZCTAs were included in the analysis. The median ZCTA-level diabetes prevalence was 12.5% (interquartile range [IQR] 10.6–14.3%), and the median rate of diabetes-related hospitalizations was 8.4 per 1000 person-years (IQR 6.0–11.4) (Table 1). The ZCTA-level median age of patients with diabetes-related hospitalizations was 67 years (IQR 64–70 years), but tended to be lower in ZCTAs with higher hospitalization rates (β = − 0.027, SE = 0.003, exp(β) = 0.974, *p* < 0.0001).

**Table 1:** Summary statistics of characteristics of ZIP code tabulation areas in Florida, 2016–2019

Theoretical domains & variables	Median	IQR^a^	n
*Health outcomes*			
Diabetes prevalence	12.5	10.6–14.3	933
DRH^b^ rate per 1000 person-years	8.4	6.0–11.4	933
Median age of DRH^b^ patients	67.0	64.0–70.0	933
Rurality/urbanization*			
Population density	471.4	83.3–1200.5	933
Rural/urban designation*			
Rural	45.4	–	424
Suburban/town	35.9	–	335
City	18.7	–	174
*Demographic characteristics*			
Median age	42.5	37.8–50.5	933
Percent 65 years of age and older	19.3	14.2–26.9	933
Percent non-Hispanic Black	8.0	3.0–17.5	933
Percent Hispanic	10.4	5.1–23.6	933
*Economic characteristics*			
Median household income	$53,988	$44,239–68,382	929
Median household value	$191,500	$135,400–274,100	919
Percent of families with income below FPL^c^	8.9	5.4–13.7	931
Percent unemployed	5.2	3.9–7.1	933
Percent of households without a vehicle	4.6	2.6–7.6	932
*Educational attainment*			
Percent aged ≥ 25 years without a high school education	10.4	6.4–16.5	933
Percent aged ≥ 25 years with a bachelor’s degree or higher	25.1	16.2–37.7	933
*Healthcare access*			
Percent without health insurance	11.7	8.4–15.7	933
Primary care physicians per 10,000 population	6.4	1.97–13.8	933
Pharmacies per 100 km^2^	13.4	1.1–52.8	933
*Built environment resources*			
Healthy food retailers per 100 km^2^	6.6	0.6–24.8	933
Less healthy food retailers per 100 km^2^	26.5	2.4–105.3	933
Modified retail food environment index	20.0	11.8–27.5	933
Recreational facilities per 100 km^2^	2.4	0–12.3	933

*Percent and frequency presented for categorical variable

^a^Interquartile range; ^b^Diabetes-related hospitalization; ^c^Federal poverty level

**Fig. 2:**
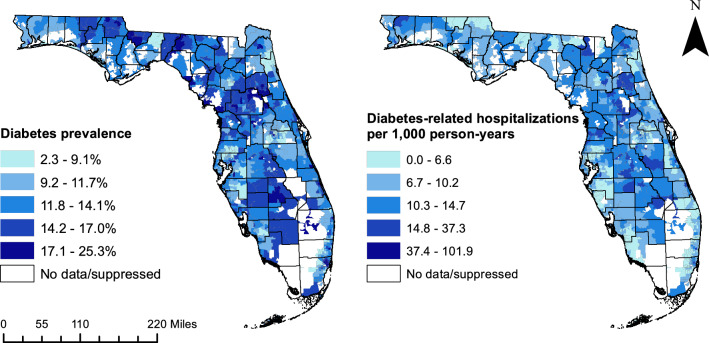
Geographic distribution of ZCTA-level diabetes prevalence estimates and smoothed diabetes-related hospitalization rates in Florida, 2016–2019

The geographic distributions of diabetes prevalence and DRH rates are displayed in Fig. 2. Rural areas, such as the region surrounding Tallahassee, north-central Florida between Orlando and Jacksonville, and south-central Florida, tended to have relatively high diabetes prevalence. In addition, there were densely populated, urban ZCTAs in Miami, Fort Lauderdale, Tampa, Orlando, and Jacksonville that had relatively high diabetes prevalence. Similarly, rural ZCTAs in north-central Florida tended to have high DRH rates, as did many of the rural ZCTAs in south-central Florida. In addition, the central urban ZCTAs of Miami, Fort Lauderdale, and Jacksonville had relatively high DRH rates. Diabetes-related hospitalization rates were comparatively lower in the suburban ZCTAs surrounding large metropolitan areas. There were some differences between the spatial patterns of diabetes prevalence and DRH rates. For example, many of the rural ZCTAs in Holmes and Jackson Counties to the east of Tallahassee had high diabetes prevalence, but relatively low rates of diabetes-related hospitalizations compared to the rest of the state.

Summary statistics of ZCTA-level characteristics are displayed in Table 1. The study area was largely comprised of ZCTAs classified as either rural (45.4%) or suburbs/towns (35.9%), with the remaining 18.7% classified as cities. The median percentage of older adults (those aged ≥ 65 years) was 19.3%. ZCTA populations had a median of 8.0% non-Hispanic Black residents, and 10.4% Hispanic residents. Median household income at the ZCTA level was $53,988, but ranged from $13,375 to $170,673. The median percent of families with income below the poverty level was 8.9%, and the median unemployment rate was 5.2%. The median percent of adults with educational attainment below the high school level was 10.4%, and a median of 25.4% of adults had a bachelor’s degree or higher. The median percent of the population without health insurance coverage at the ZCTA level was 11.7%, but ranged from 0% to 50.4%. Built environment characteristics such as density of pharmacies, food retailers, and recreational centers were highly correlated (|*r*_*s*_|> 0.7) with one another, and pharmacy and food retailer density were also highly correlated with population density and rural–urban classification.

### Predictors of diabetes-related hospitalization rates

#### Global ordinary least squares regression model

Results of the final global multivariable ordinary least squares regression model are displayed in Table 2. Explanatory variables that had statistically significant, positive associations with log-transformed diabetes-related hospitalization rates included the percent of the population aged 65 and older (*p* < 0.0001), percent of non-Hispanic Black residents (*p* = 0.003), unemployment (*p* = 0.001), percent without health insurance coverage (*p* < 0.0001), and percent of households without access to a vehicle (*p* = 0.002). Population density (*p* < 0.0001) and median household income (*p* < 0.0001) had significant negative associations with DRH rates. Since ethnicity and educational attainment have previously been identified as predictors of self-management behaviors, access to healthcare, and diabetes-related outcomes in both individual-level and ecological studies, the percent of Hispanic residents and percent without a high school education were retained in the model despite their lack of statistically significant associations with the outcome [9, 16, 61–71]. No evidence of confounding was identified based on percent change in coefficient estimates during backwards elimination.

**Table 2:** Predictors of log-transformed ZCTA-level diabetes-related hospitalization rates in Florida, 2016–2019

Parameter	β^a^ (SE^b^)	exp(β^a^)	*p*-value	VIF^c^
Intercept	− 0.010 (0.023)	0.990	0.664	0
Population density	− 0.240 (0.031)	0.787	< 0.0001	1.850
Percent 65 and older	0.125 (0.027)	1.133	< 0.0001	1.436
Percent non-Hispanic Black	0.085 (0.029)	1.089	0.003	1.636
Percent Hispanic	− 0.020 (0.031)	0.980	0.518	1.879
Median household income	− 0.458 (0.034)	0.633	< 0.0001	2.242
Percent without a high school education	0.041 (0.036)	1.042	0.253	2.535
Unemployment rate	0.092 (0.027)	1.096	0.001	1.207
Percent without health insurance	0.118 (0.032)	1.125	< 0.0001	1.962
Percent without access to a vehicle	0.097 (0.032)	1.102	0.002	2.020

^a^Regression coefficient; ^b^Standard error; ^c^Variance inflation factor

**Fig. 3:**
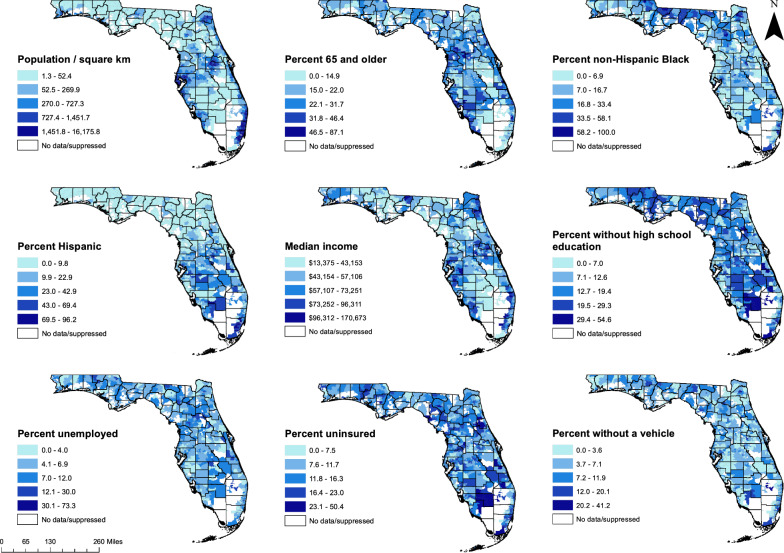
Geographic distribution of predictors of ZCTA-level diabetes-related hospitalization rates in Florida, 2016–2019

The geographic distributions of the predictor variables included in the global OLS model are displayed in Fig. 3. Populations of ZCTAs along the Gulf Coast, south of the Tampa Bay area, and in the rural region to the north of Tampa and northeast of Orlando had the highest percentages of adults aged 65 and older, while the percentage of older adults in populations of major cities was relatively low. The percent of non-Hispanic Black residents tended to be highest in populations of ZCTAs surrounding Tallahassee, as well as in large cities such as Jacksonville, Orlando, Miami, and Fort Lauderdale. The percent of Hispanic residents was lowest in populations of ZCTAs in northern Florida and the panhandle, and tended to be higher in central and south Florida. There were similarities in the distribution of several socioeconomic variables, including median household income, the unemployment rate, vehicle access, and educational attainment. Suburban and fringe rural ZCTAs adjacent to urban areas tended to have higher income, educational attainment, employment rates, and vehicle access, while these were often lower in distant or remote rural areas as well as some of the most densely populated urban ZCTAs in major cities such as Miami and Jacksonville.

#### Multiscale geographically weighted regression model

Results of the multiscale geographically weighted regression model are displayed in Tables 3, 4, 5. The interquartile ranges of the local regression coefficients in the MGWR model were greater than twice the standard errors of the coefficients from the global model for all parameters except the percent of Hispanic residents (Table 3). These findings indicate that the coefficients for the intercept and all other explanatory variables in the model exhibited significant spatial non-stationarity, varying in strength based upon geographic location in the study area.

**Table 3:** Assessment of spatial non-stationarity of regression coefficients in MGWR model predicting log-transformed diabetes-related hospitalization rates

Parameter	SE^a^ (OLS^b^)	IQR^c^ (MGWR^d^)	IQR^c^–2(SE^a^)
Intercept	0.023	0.489	0.443
Population density	0.031	0.287	0.225
Percent 65 and older	0.027	0.190	0.136
Percent non-Hispanic Black	0.029	0.257	0.199
Percent Hispanic	0.031	0.010	− 0.052
Median household income	0.034	0.157	0.089
Percent without a high school education	0.036	0.362	0.290
Unemployment rate	0.027	0.177	0.123
Percent without health insurance	0.032	0.343	0.279
Percent without access to a vehicle	0.032	0.108	0.044

^a^Standard error; ^b^Global ordinary least squares regression model; ^c^Interquartile range; ^d^Multiscale geographically weighted regression model

**Table 4:** Summary of MGWR model predicting log-transformed ZCTA-level diabetes-related hospitalization rates

Parameter	Bandwidth	ENP^a^	Critical *t*-value	Number (%) of ZCTAs with significant coefficients
Intercept	25	83.657	3.445	148 (15.9)
Population density	315	4.052	2.507	739 (79.5)
Percent 65 and older	146	10.034	2.815	763 (82.1)
Percent non-Hispanic Black	27	63.952	3.370	111 (12.8)
Percent Hispanic	920	1.114	2.009	0 (0)
Median household income	275	5.040	2.584	739 (79.5)
Percent without a high school education	54	30.521	3.158	207 (22.3)
Unemployment rate	199	7.805	2.732	207 (22.3)
Percent without health insurance	81	20.167	3.034	301 (32.4)
Percent without access to a vehicle	446	3.703	2.475	421 (45.3)

^a^Effective number of parameters

**Table 5:** Distribution of local coefficients from MGWR model predicting log-transformed ZCTA-level diabetes-related hospitalization rates

Parameter	Local coefficients		
	Minimum	Median	Maximum
Intercept	− 0.713	0.038	0.756
Population density	− 0.577	− 0.244	− 0.093
Percent 65 and older	0.022	0.294	0.590
Percent non-Hispanic Black	− 1.035	0.186	1.118
Percent Hispanic	− 0.006	0.008	0.013
Median household income	− 0.449	− 0.341	− 0.057
Percent without a high school education	− 0.355	0.195	0.649
Unemployment rate	− 0.112	0.068	0.234
Percent without health insurance	− 0.170	0.075	0.714
Percent without access to a vehicle	0.011	0.075	0.188

Since the bandwidths in an MGWR model control the number of local observations (i.e. number of nearest neighbors) used to compute regression coefficients, their values indicate the spatial scale at which the associations between explanatory variables and the outcome vary within the study area [53–55]. A variable with a small bandwidth relative to the number of units in the study area has a large effective number of parameters (ENP) and greater spatial heterogeneity, while a bandwidth approaching the total number of observations and ENP close to 1 indicate little spatial variation in the strength of the association [53–55]. The optimal bandwidths identified in this study varied, suggesting that simpler standard or semiparametric GWR models would not adequately describe the spatial variation in the observed associations (Table 4). Comparison of the AICc values from the global OLS model (AICc = 1951.469) and the MGWR model (AICc = 1414.473) indicated that the MGWR model had better fit to the data than the global model.

**Fig. 4:**
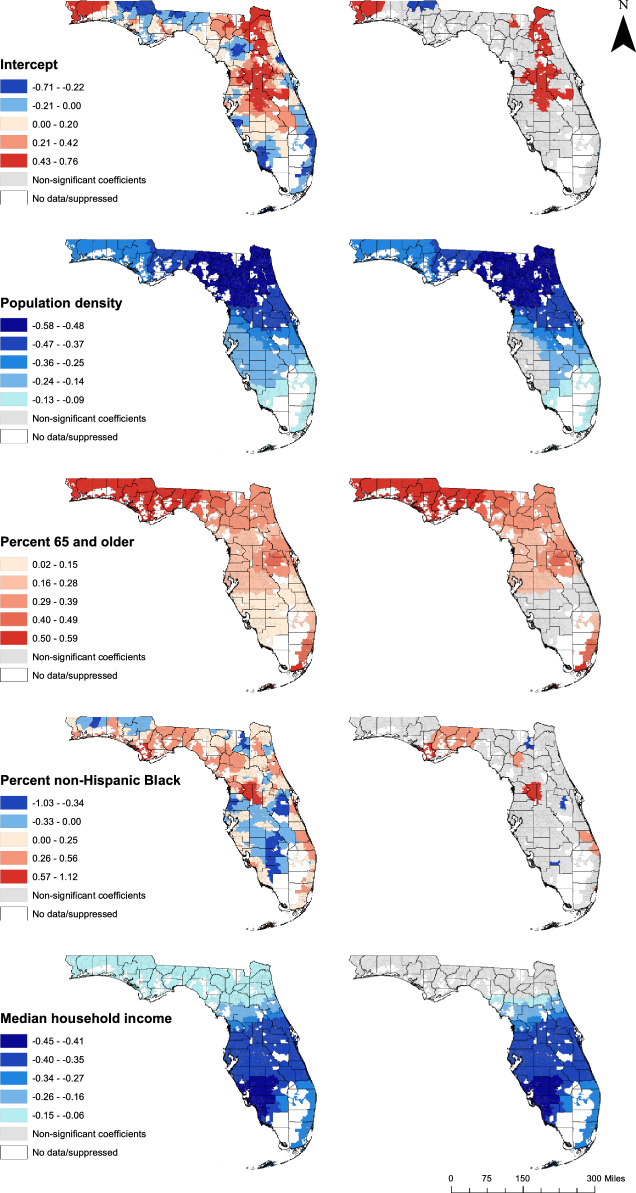
Distribution of all (left) and significant (right) local coefficients of predictors of diabetes-related hospitalization rates

**Fig. 5:**
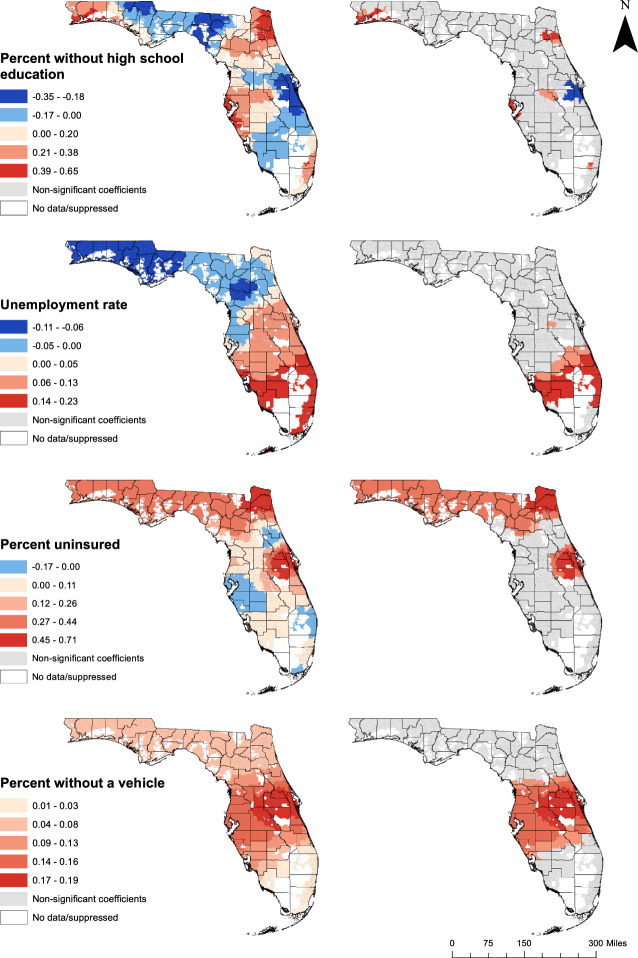
Distribution of all (left) and significant (right) local coefficients of predictors of diabetes-related hospitalization rates

The geographic distributions of coefficient estimates for explanatory variables with significant spatial non-stationarity from the final MGWR model are presented in Figs. 4 and 5. For each model parameter, the map on the left shows the distribution of coefficient estimates for all ZCTAs in the study area, while the map on the right shows only statistically significant estimates, with correction for multiple hypothesis testing. Parameter estimates with the lowest bandwidths relative to the number of units in the study area, implying regression coefficients with the most spatial heterogeneity, included the intercept (BW = 25), percent of non-Hispanic Black residents (BW = 27), and educational attainment (BW = 54) (Table 4). This spatial heterogeneity is apparent in the maps of the regression coefficients, which exhibited a great deal of local variation and were only significant in a subset of ZCTAs in the study area (Figs. 4 and 5).

Another subset of explanatory variables had associations with DRH rates that varied at a more regional spatial scale, as evidenced by their bandwidth values and the geographic distribution of their regression coefficients. These included population age composition (BW = 146), the unemployment rate (BW = 199), and percent without health insurance coverage (BW = 81) (Table 4). The percent of adults aged 65 and older was significantly associated with DRH rates in 82.1% of the ZCTAs in the study area, with the strongest associations in the panhandle, the Orlando area, and south of Fort Lauderdale (Fig. 4). Significant associations between the unemployment rate and DRH rates occurred primarily in southern Florida, and were also observed in southwest Orange County, including part of the Orlando area (Fig. 5). Health insurance coverage was significant predictor of DRH rates in about one-third (32.4%) of ZCTAs, with the strongest associations in the Jacksonville and Orlando areas (Fig. 5).

Non-stationary variables with the largest bandwidths, implying less heterogeneity of regression coefficients, included population density (BW = 315), median income (BW = 275), and vehicle access (BW = 446) (Table 4). The variation in the strengths of their associations with DRH rates occurred at a larger spatial scale, with less heterogeneity within the study area. For instance, the negative association between population density and DRH rates, which was significant in most of the study area, was strongest in northern Florida and gradually decreased in magnitude toward the southern and western parts of the state (Fig. 4). Median household income was also negatively associated with DRH rates in most of the state, with the strongest associations observed in Southwest Florida (Fig. 4). The percent of households that lacked access to a vehicle was a significant predictor of DRH rates across central Florida (Fig. 5).

## Discussion

This study identified determinants of local (ZCTA-level) geographic disparities of diabetes-related hospitalization rates in Florida. Identified significant determinants included population demographic composition, socioeconomic characteristics, and rurality. This study also demonstrated the use of multiscale geographically weighted regression as a tool for describing the spatial variation in the associations between these determinants and DRH rates. The observed associations differed in both strength and direction based on geographic location within the state, and exhibited differing levels of spatial heterogeneity.

Populations with high DRH rates tended to have higher proportions of older adults, an association that was significant in most of the state. At the individual level, age is a risk factor for diabetes as well as macrovascular complications such as stroke and myocardial infarction, so this finding was not surprising [72, 73]. To some extent, the positive association between DRH rates and the percent of adults over the age of 65 may also reflect utilization of hospital services, because this population has fewer gaps in health insurance coverage due to Medicare eligibility [74, 75]. The strength and statistical significance of the association between population age composition and DRH rates varied based upon geographic location. Weak and/or no associations, particularly in areas with relatively high incomes and percentages of older adults, may, in part, reflect the presence of active retirement communities and self-selecting populations of healthy older adults. It is also worth noting that earlier age of onset of type 2 diabetes is a predictor of adverse diabetes-related outcomes, independent of duration of disease [9, 76–78], and patients with diabetes-related hospitalizations in this study tended to be younger in areas with high DRH rates, consistent with previous research [71]. Thus, it is possible that the association between population age composition and DRH rates was attenuated in some parts of the state, particularly those with disproportionately high DRH rates, due to the elevated risk profile of younger patients with diabetes.

Significant, ZCTA-level associations between household income and DRH rates were observed across most of the study area. This finding may be due to a combination of individual- and area-level processes, and is consistent with previous research. Patients with diabetes who report lower income levels are more likely to forego medical care due to cost, and less likely to receive routine HbA1c testing, cholesterol testing, and eye examinations [16, 68, 79]. Furthermore, neighborhood-level income and poverty are associated with heart disease, renal disease, and lower extremity amputations [80, 81], and perceived neighborhood safety is a predictor of treatment nonadherence among adults with diabetes [82]. County-level associations between socioeconomic status and the burden of diabetes complications and ambulatory care sensitive hospitalizations have also been reported in the US [83–85].

In this study, significant local associations between median household income and DRH rates were not observed in northern Florida and the panhandle. Median income tended to be low in many of the ZCTAs in this region, with a more homogenous distribution than that of central and southern Florida. Therefore, the observed finding may, at least in part, be due to a lack of variability in median income between ZCTAs in this region. Interestingly, most of the ZCTAs with significant local associations between insurance coverage and diabetes-related hospitalizations were located in this area of northern Florida, where income was not significantly associated with DRH rates. The observed association between health insurance coverage and DRH rates is not surprising; at the individual level, health insurance is a predictor of linkage to care, diagnostic testing, screening for complications, and glycemic control among patients with diabetes [9, 68, 79]. Furthermore, patients without health insurance are at higher risk of diabetes-related hospital deaths than those with insurance [17]. The regional variation in the magnitude of local coefficients for health insurance coverage was consistent with findings of a study that used MGWR to investigate determinants of mortality rates in the US [86]. This regional variation could reflect differences in the availability of resources for uninsured individuals within the state, such as county health programs and services and/or community health centers [87]. For instance, health insurance coverage could be a more important determinant of potentially avoidable hospitalization rates in areas with fewer providers of low-cost primary care services. In some areas, access to affordable services may be a more relevant predictor of receipt of preventive care than the supply of primary care physicians. This could explain the observed lack of association between DRH rates and primary care physicians per capita in this study. The finding that income was a stronger determinant of DRH rates than insurance coverage in much of the state may also reflect cost barriers to healthcare access and effective self-management experienced by many patients despite having health insurance coverage. While the underlying cause of the observed spatial heterogeneity remains to be determined, findings of this study suggest that efforts to expand health insurance coverage and access to affordable care for those without coverage will be particularly important in the regions where insurance was a significant determinant of DRH rates.

Population-level educational attainment and unemployment were associated with DRH rates in smaller subsets of the study area after controlling for income and the other variables in the model. Among those with diabetes, educational attainment below the high school level has previously been associated with healthcare quality and utilization in global models [16, 62, 66]. Educational attainment and unemployment may also be associated with other factors such as income and health insurance [87], which may explain the lack of association observed in many parts of the study area.

Population density, which was used as a measure of rurality in this study, was significantly associated with DRH rates, with more densely populated areas tending to have lower rates of hospitalizations. In the present study, the association between population density and DRH rates was strongest in northern Florida, where spatial clusters of pre-diabetes and diabetes prevalence [20, 22, 88], as well as stroke prevalence [89] and myocardial infarction mortality [90] have been identified. Previous research suggests that rural residents are more likely to report delaying health care due to cost than urban residents [91]. Furthermore, there is evidence that quality of care is worse for rural patients with diabetes than for those living in metropolitan areas [68]. In Florida, rural populations also tend to have lower rates of participation in diabetes self-management education programs [21]. In the current study, ZCTAs along the south-central Gulf Coast of Florida that did not have significant associations between population density and DRH rates had some of the strongest relationships between this outcome and median income. This could suggest that rural–urban disparities are driven by socioeconomic disparities in some areas; however, further research is necessary to support this hypothesis.

It is worth noting that population density was selected from a group of highly correlated variables, which included pharmacy and food retailer densities, for inclusion in the modeling process. Thus, the observed association between rurality and DRH rates, particularly in northern Florida, may reflect the availability of built environment resources to some extent. Several counties in northern Florida are part of the Diabetes Belt [13, 14], where predictors of diabetes prevalence were reported to differ from the rest of the United States [92]. These included recreational facility density and natural amenities, predictors that were unique to the Diabetes Belt [92].

Transportation barriers may also contribute to rural–urban disparities in health outcomes by creating additional time demands for patients. Patients who experience transportation barriers to health care tend to utilize emergency department services more frequently and tend to have worse self-rated health status compared to those who do not report such barriers [91, 93]. A review of transportation barriers to healthcare access found that access to a vehicle was consistently identified as a predictor of access to care, while there was less evidence in support of distance to providers as a barrier to receiving care [94]. This is in line with the findings of our study, which identified vehicle access, but not density of primary care physicians, as a significant predictor of diabetes-related hospitalization rates. Regional differences in transportation barriers to health care have previously been reported in the US, with residents of the South and Midwest being more likely to report such barriers than those from other parts of the country [93]. The geographic variation in the association between vehicle access and DRH rates observed in the present study could reflect differences in the availability of alternate forms of transportation. Vehicle access was a significant determinant of DRH rates in central Florida in a region that included both rural and urban ZCTAs. Interestingly, however, significant local associations were not observed in northern Florida, which is largely rural, or in southern Florida. In some areas, residents of urban areas may also face transportation barriers to healthcare access; a North Carolina study reported that the odds of reporting delayed care due to transportation difficulties did not differ between older adults from rural and urban counties [91]. Concerns with safety, cost, and convenience may affect the use of public transportation to access healthcare services and health-promoting resources, which may contribute to the relevance of vehicle access in urban as well as rural areas [94].

Despite controlling for population-level socioeconomic characteristics, the percent of non-Hispanic Black residents remained a significant predictor of DRH rates in certain parts of the state, with most of these associations being positive. At the individual level, previous studies have reported that Black patients with diabetes are less likely than White patients to receive recommended diagnostic testing and examinations [66, 68, 79] and more likely to forego necessary medical care due to cost [16]. Furthermore, non-Hispanic Black patients with diabetes are less likely to achieve diabetes management targets [9], and tend to have more severe complications [64]. Ecological studies in the US, which have investigated burdens of end-stage renal disease, stroke, and diabetes-related lower extremity amputations, have reported similar findings [80, 83, 85]. The degree of spatial heterogeneity in the association observed in the current study highlights the importance of locally-focused interventions to close gaps in access, quality, and utilization of diabetes-related care in order to reduce inequities in diabetes outcomes.

The map of significant local intercept parameter estimates from the MGWR model indicates that there are elevated DRH rates, mostly in ZCTAs in the central and northeastern part of the state, as well as a few ZCTAs in the panhandle, with lower DRH rates than expected after controlling for the explanatory variables in the model. In the central and northeast Florida ZCTAs that had significant, positive intercept parameter estimates, there may be additional drivers of excess DRH rates that were not identified in this study.

### Strengths and limitations

To our knowledge, this is the first study to investigate determinants of geographic disparities of diabetes-related hospitalization rates at the sub-county level using multiscale geographically weighted regression. Multiscale geographically weighted regression provides a more detailed understanding of the spatial heterogeneity of these associations than standard GWR, which assumes that all processes operate at the same spatial scale within the study area [53–57]. Understanding the spatial context of the observed relationships provides valuable insights to guide health planning aimed at improving diabetes management and outcomes at the population level. However, this study is not without limitations. While populations of ZCTAs that did not meet the inclusion criteria were similar to those included in the study in terms of age, gender, and income, they differed with respect to characteristics such as racial composition, health insurance coverage, and educational attainment (Table S1). Therefore, study findings may not be generalizable to populations of excluded ZCTAs. In addition, relatively small sample sizes in some ZCTAs may have reduced statistical power to detect significant local associations, particularly for variables that had a high degree of spatial heterogeneity and large effective number of parameters. Models predicting diabetes-related hospitalization rates were not adjusted for underlying diabetes prevalence, comorbidities, or health behaviors, since direct estimates of these measures are not available at the ZCTA level, and the use of small area estimates could have introduced bias into the analysis [95]. Despite these limitations, the findings of this study are useful for guiding evidence-based interventions by identifying determinants of diabetes-related hospitalization rates, which have been shown to reflect population-level glycemic control [96].

## Conclusions

Addressing barriers to diabetes-related care and effective management of the condition, particularly in communities disproportionately burdened by adverse outcomes, is essential for reducing disparities. The findings of this study underscore the importance of socioeconomic resources and rurality in shaping population health. In addition, this study highlights the usefulness of multiscale geographically weighted regression as a tool for investigating spatially variable determinants of diabetes-related hospitalizations, which exhibited different levels of heterogeneity within the study area. Knowledge of the determinants most strongly associated with DRH rates in a given location can help guide the development of needs-based policies and interventions that address barriers to achieving diabetes care targets in order to reduce the burden of potentially avoidable hospitalizations.

### Supplementary Information

**Additional file 1. Table S1.** Characteristics of included vs. excluded diabetes-related hospitalizations and ZIP code tabulation areas in Florida, 2016-2019.

## Data Availability

Restrictions apply to the availability of the hospital discharge data used in this study, which were provided by the Florida Agency for Health Care Administration through a Data Use Agreement with the Florida Department of Health. Details of the data and how to request access are available from the Florida Agency for Health Care Administration (https://quality.healthfinder.fl.gov/Researchers/OrderData/order-data.aspx).

## Abbreviations

AHRQ: Agency for Healthcare Research and Quality
AICc: Bias-corrected Akaike’s information criterion
BW: Bandwidth
CDC: Centers for Disease Control and Prevention
DCSI: Diabetes Complications Severity Index
DRH: Diabetes-related hospitalization
ENP: Effective number of parameters
GWR: Geographically weighted regression
HbA1c: Hemoglobin A1c
IQR: Interquartile range
MGWR: Multiscale geographically weighted regression
mRFEI: Modified retail food environment index
NAICS: North American Industry Classification System
OLS: Ordinary least squares
PLACES: Population-Level Analysis and Community Estimates
SAS: Statistical Analysis System
TIGER: Topologically Integrated Geographic Encoding and Referencing
US: United States
VIF: Variance inflation factor
ZCTA: ZIP code tabulation area
ZIP: Zone improvement plan
