# Supplementary material for: Determinants of disparities of diabetes-related hospitalization rates in Florida: a retrospective ecological study using a multiscale geographically weighted regression approach
Source: Int J Health Geogr. 2024 Jan 6;23:1. doi: 10.1186/s12942-023-00360-5 (PMC10771651; doi:10.1186/s12942-023-00360-5)
Supplement: Supplementary file 1 — Additional file 1. Table S1. Characteristics of included vs. excluded diabetes-related hospitalizations and ZIP code tabulation areas in Florida, 2016-2019. [file 12942_2023_360_MOESM1_ESM.docx]

**Appendix Table 1. Characteristics of included vs. excluded diabetes-related hospitalizations and ZIP code tabulation areas in Florida, 2016-2019.**

| **Variable name** | **Study ZCTAs**^1^ | | **Excluded ZCTAs**^1^ | | ***p*-value**^3^ |
| --- | --- | --- | --- | --- | --- |
|  | **Median (IQR**^2^**)** | **n** | **Median (IQR**^2^**)** | **n** |  |
| ***Patient-level variables*** |  |  |  |  |  |
| Age | 67 | 554,133 | 67 | 546 | 0.1633 |
| Gender* |  |  |  |  | 0.9136 |
| Male | 57.8 | 320,450 | 58.1 | 317 |  |
| Female | 42.2 | 233,683 | 41.9 | 229 |  |
| Race/ethnicity* |  |  |  |  | < 0.0001 |
| Non-Hispanic White | 59.0 | 321,260 | 71.4 | 382 |  |
| Non-Hispanic Black | 20.7 | 112,696 | 14.8 | 79 |  |
| Hispanic | 17.6 | 95,778 | 9.2 | 49 |  |
| Other Non-Hispanic | 2.7 | 14,477 | 4.7 | 25 |  |
| Principal payer* |  |  |  |  | 0.0023 |
| Medicare | 63.7 | 353,027 | 64.8 | 354 |  |
| Medicaid | 10.2 | 56,330 | 9.7 | 53 |  |
| Private | 15.4 | 85,746 | 15.0 | 82 |  |
| VA, TriCare, other government | 2.6 | 14,533 | 5.1 | 28 |  |
| Self-pay (uninsured) | 5.9 | 32,526 | 4.0 | 22 |  |
| Non-payment/Other | 2.2 | 12,241 | 1.3 | 7 |  |
| ***ZCTA-level variables*** |  |  |  |  |  |
| *Rurality/urbanization* |  |  |  |  |  |
| Population density | 471.4 (83.3 - 1200.5) | 933 | 54.5 (3.7 - 299.8) | 50 | < 0.0001 |
| Rural/urban designation* |  |  |  |  | 0.0015 |
| Rural | 45.4 | 424 | 72.0 | 36 |  |
| Suburban/town | 35.9 | 335 | 16.0 | 8 |  |
| City | 18.7 | 174 | 12.0 | 6 |  |
| *Demographic characteristics* |  |  |  |  |  |
| Median age | 42.5 (37.8 - 50.5) | 933 | 43.9 (21.8 - 57.5) | 39 | 0.2887 |
| Percent 65 years of age and older | 19.3 (14.2 - 26.9) | 933 | 11.0 (0 - 35.0) | 42 | 0.0263 |
| Percent non-Hispanic Black | 8.0 (3.0 - 17.5) | 933 | 2.2 (0 - 10.6) | 42 | < 0.0001 |
| Percent Hispanic | 10.4 (5.1 - 23.6) | 933 | 12.8 (0 - 19.5) | 42 | 0.1027 |
| *Economic characteristics* |  |  |  |  |  |
| Median household income | $53,988 ($44,239 - 68,382) | 929 | $52,321 ($39,038 - 61,442) | 17 | 0.5928 |
| Median household value | $191,500 ($135,400 - 274,100) | 919 |  |  |  |
| Percent of families with income below FPL | 8.9 (5.4 - 13.7) | 931 | 0 (0 - 26.3) | 33 | 0.0059 |
| Percent unemployed | 5.2 (3.9 - 7.1) | 933 | 0 (0 - 1.5) | 37 | < 0.0001 |
| Percent of households without a vehicle | 4.6 (2.6 - 7.6) | 932 | 0 (0 - 1.1) | 36 | < 0.0001 |
| *Educational attainment* |  |  |  |  |  |
| Percent aged ≥25 years without a high school education | 10.4 (6.4 - 16.5) | 933 | 2.8 (0 - 19.4) | 41 | 0.0014 |
| Percent aged ≥25 years with a bachelor’s degree or higher | 25.1 (16.2 - 37.7) | 933 | 20.0 (0 - 28.0) | 41 | 0.0002 |
| *Healthcare access* |  |  |  |  |  |
| Percent without health insurance | 11.7 (8.4 - 15.7) | 933 | 7.4 (0 - 17.4) | 37 | 0.0089 |
| Primary care physicians per 10,000 population | 6.4 (1.97 - 13.8) | 933 | 0 (0 - 0) | 44 | < 0.0001 |
| Pharmacies per 100 km^2^ | 13.4 (1.1 - 52.8) | 933 | 0 (0 - 0) | 50 | < 0.0001 |
| *Built environment resources* |  |  |  |  |  |
| Healthy food retailers per 100 km^2^ | 6.6 (0.6 - 24.8) | 933 | 0 (0 - 0) | 50 | < 0.0001 |
| Less healthy food retailers per 100 km^2^ | 26.5 (2.4 - 105.3) | 933 | 0 (0 - 11.2) | 50 | < 0.0001 |
| Modified retail food environment index | 20.0 (11.8 - 27.5) | 933 | 0 (0 - 0) | 50 | < 0.0001 |
| Recreational facilities per 100 km^2^ | 2.4 (0 - 12.3) | 933 | 0 (0 - 0) | 50 | < 0.0001 |

*Percent for categorical variables

^1^ZIP code tabulation areas

^2^Interquartile range

^3^*p*-value for Chi-square test for categorical variables, Wilcoxon rank sum test for continuous variables
